# Understanding MeJA induced-resistance to *Phytophthora cinnamomi* in holm oak embryogenic lines

**DOI:** 10.3389/fpls.2025.1740888

**Published:** 2026-02-02

**Authors:** Marian Morcillo, Rosa Sanchez-Lucas, Ester Sales, Jesús Jorrín-Novo, Eva Miedes, Isabel Arrillaga

**Affiliations:** 1 Plant Biology Department, Biotechnology and Biomedicine (BiotecMed) Institute, Universitat de València, Burjassot, Valencia, Spain; 2 Agroforestry and Plant Biotechnology (AGR-164) Department of Biochemistry and Molecular Biology, University of Cordoba, Cordoba, Spain; 3 Agrarian and Environmental Sciences Department, Institute for Research on Environmental Sciences (IUCA), High Polytechnic School, University of Zaragoza, Huesca, Spain; 4 Biotechnology - Plant Biology Department, Escuela Técnica Superior de Ingeniería Agronómica, Alimentaria y de Biosistemas, Universidad Politécnica de Madrid, Madrid, Spain

**Keywords:** agroecosystem, elicitor, nut crops, oak decline, plant pathogens, priming, transgenerational memory, untargeted proteomics

## Abstract

Holm oak (*Quercus ilex* L.) decline in Mediterranean forests is mostly driven by the synergistic effects of drought and *Phytophthora cinnamomi*, yet effective protection strategies remain elusive. Previously, we reported that elicitation of holm oak embryogenic lines with 50 µM methyl jasmonate (MeJA), did not impair somatic embryo growth and development while inducing jasmonic acid (JA) and phenolic compounds accumulation, also resulting in increased H_2_O_2_ levels and further JA production after challenged against active oomycete mycelium. Here, we evaluate the proteomic profile in the E00 embryogenic line of *Q. ilex* in response to this priming treatment and subsequent pathogen inoculation. To this end, embryogenic cultures were first treated with a solution of 0 or 50 µM MeJA for three days and five days later, inoculated or not with a *P. cinnamomi* mycelia extract. Twenty-four hours post-inoculation, samples were harvested, proteins extracted from the four treatments, and analysed by nano-LC-MS/MS on an Orbitrap Fusion. SEQUEST searches against a translated *Q. ilex* transcriptome identified 3,205 protein species with high confidence. Multivariate sparse PLS-DA captured 68.9% of variance and clearly discriminated between treatments, particularly separating inoculated from non-inoculated samples. MeJA elicitation led to the accumulation of proteins associated with cell-wall biogenesis, carbon metabolism, and amino-acid and phenylpropanoids biosynthesis. Some of these proteins were also accumulated, but to a lesser extent, by pathogen inoculation in non-elicited cultures. Notably, 24 hours after inoculation, MeJA – elicited samples showed enhanced abundance of proteins hormone-signalling and redox enzymes, including cinnamate-4-hydroxylase, caffeoyl-CoA O-methyltransferase, glutathione S-transferases, calreticulin, thaumatin-like proteins, catalase and chitinase. These results revealed that MeJA elicitation induced a primed state in *Q. ilex* embryogenic lines, reprograming the proteome to enhance early defence and oxidative stress response pathways upon *P. cinnamomi* e inoculation. This supports MeJA priming as a promising biotechnological strategy to improve holm oak resilience and productivity complementing integrated pest management and breeding strategies.

## Graphical Abstract

**Figure:**
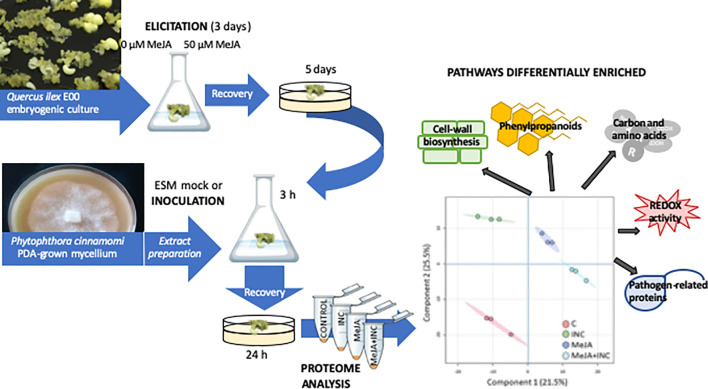

## 1 Introduction

Natural forest ecosystems of the Mediterranean Basin are dominated by holm oak trees (*Quercus ilex* L). This species, along with cork oak (*Q. suber* L), is also a vital component of the “*dehesas*” or “*montados*” agroecosystem in the Iberian Peninsula. Holm oak is mainly used to provide acorns for animal feeding and for black truffle production but, lately, acorns nutritional value, its high contents in phytochemical compounds with biological activity (such as antioxidant, anticarcinogenic, and cardioprotective properties), have raised the interest in integrating these fruits into the human diet (Vinha et al., 2016). In recent years, however, populations of many oak species, and especially those of holm oak, have dramatically declined in Portugal, Italy and Spain, where tree mortality has significantly increased (Català et al., 2017).

Holm oak decline is a complex syndrome that is manifested by gradual crown de-foliation, loss of tree vigour and, finally, death of the trees (Vivas et al., 2021). The etiology of the disease is multiple and usually results from the interaction of several factors (Moreno-Fernández et al., 2019), such as aging and low natural regeneration of trees (Ruiz-Gómez et al., 2019), and the impact of extreme climate events promoted by climate change, such as increased temperatures and altered precipitation regimes (Gea-Izquierdo et al., 2021; Maldonado-Alconada et al., 2022; Sena et al., 2018), and the appearance of new diseases and outbreaks of soil pathogens, mainly *Phytophthora* spp (de Sampaio e Paiva Camilo-Alves et al., 2013; Morcillo et al., 2020). Studies on oaks infection by *Phytophthora* reported that although trees have been infected 50 years ago, water stress episodes can act synergistically at different spatial-temporal scales to produce tree death (Gea-Izquierdo et al., 2021). However, tolerance of holm oak against *Phytophthora cinnamomi* root rot is related to specific hydric and photosynthetic mechanisms that differ from those associated with drought (Ruiz Gómez et al., 2018).

The economic and social values of the biodiversity in this forest ecosystem (Ramírez-Hernández et al., 2014) have raised many efforts to study causes of the symptoms of the oak decline syndrome, and also to develop strategies that help to adopt good management practices (Martínez et al., 2022). Key requirements for successful prediction, control and management of the holm oak decline have been recently reviewed (Ruiz-Gómez et al., 2019). These include a thorough knowledge of the cellular and molecular basis of the pathogen’s biology and pathogenicity (Hardham and Blackman, 2018), as well as methods for its rapid and sensitive detection and identification (Sena et al., 2018), and multifaceted control procedures including the use of beneficial microorganisms such as *Trichoderma* spp (Corcobado et al., 2014; Ruiz-Gómez et al., 2019). Multiomics and molecular approaches to identify markers associated to *Quercus* species tolerance to abiotic and biotic stresses have been also reviewed (Escandón et al., 2021; Maldonado-Alconada et al., 2022; Triviño et al., 2025; Alcaide et al., 2025). The tools and knowledge derived from the molecular approach might help to select tolerant genotypes that, after clonal propagation, can be used in reforestation, conservation, and improvement programs, therefore contributing to mitigate the effect of climate change (Escandón et al., 2021).

Among the available biotechnological tools, somatic embryogenesis may be a valuable option regarding the propagation and conservation of selected holm oak genotypes (Martínez et al., 2021; 2022). Also, somatic embryogenesis protocols have been proved to be an excellent system to study the use of elicitors in this forest tree species (Morcillo et al., 2020; 2022; Ruiz-Galea et al., 2023). Pre-exposure of plants, seeds or somatic embryos, to an eliciting factor enables primed somatic embryos or plants to be more tolerant to later biotic or abiotic stress events. Priming induced an “stress memory” that exists in both the present generation and the offspring. Thus, priming is suggested to be a promising strategy for plants to cope with the biotic and abiotic stresses under global change scenarios (Liu et al., 2022). Although most studies of transgenerational induction of defences to pests and pathogens in plants have focused on short-lived annuals, recent evidences support the fact that transgenerational plasticity also occurs in long-lived forest trees (Morcillo et al., 2020). For instance, external application of methyl jasmonate (MeJA) increased the production of resin-based defences in mature *P. pinaster* trees for at least two years after its application (Vázquez-González et al., 2022), and Camisón et al. (2019) demonstrated that chestnut (*Castanea sativa*) seedlings of ink-diseased mother trees showed increased tolerance to the *P. cinnamomi* the causing agent of this disease. Interestingly, this tolerance was not mediated by seed size, but probably as a consequence of seed priming during fruit development. Similarly, transgenerational-induced resistance to *P. cinnamomi* has been recently reported in *Q. ilex* seedlings (Vivas et al., 2021). Recently it was reported that oomycete protection in chesnut seedlings, induced by a preventive MeJA spray, lasted one-year (Dorado et al., 2025).

The first evidence of transgenerational- induced resistance using somatic embryos in forest tree species was reported by Kvaalen and Johnsen (2008) demonstrating that temperature changes during somatic embryogenesis of Norway spruce affect phenological characteristic in their derived plants. More recently, our group demonstrated that applying high temperatures during somatic embryogenesis induction (Pérez-Oliver et al., 2021) or varying temperatures during maritime pine somatic embryo maturation (Sales et al., 2022) produced plants with better adaptation to heat stress. Furthermore, holm oak somatic embryos were used to study the potential of chemical elicitation treatments to induce defence responses to *Phytophthora cinnamomi* (Morcillo et al., 2020). Among several chemical elicitors, MeJA was proposed as an induced resistance (IR) stimulus (Morcillo et al., 2022) on the bases of the altered hormonal and phenolic profiles.

The regulatory mechanisms of plants in response to elicitation treatment and biotic stress can be studied by proteomic approaches (Lemaître-Guillier et al., 2017), that have great potential in plant biological research in general, and in the genus *Quercus* in particular, and that have been little exploited thus far. Several proteomic studies related to immune response of cork and holm oak to *Phytophthora* inoculation have been reported (Sghaier-Hammami et al., 2013; Coelho et al., 2021), while Escandón et al. (2021) reported that holm oak proteomic profile varies similarly after a biotic stress and after inoculation with the oomycete. Despite these recent findings, much is still unknown about the molecular mechanisms underpinning the establishment and maintenance of MeJA-IR in holm oak. Here we present and discuss changes in the proteomic profile after priming an embryogenic line with MeJA and further inoculation with *P. cinnamomi*.

## 2 Materials and methods

### 2.1 Plant and oomycete material

The holm oak E00 embryogenic line used in this study was generated from immature acorns as described in Barra-Jiménez et al. (2014), and kindly provided by Dr. M. Toribio from IMIDRA (Instituto Madrileño de Investigación y Desarrollo Rural, Agrario y Alimentario, Madrid, Spain). E00 was routinely maintained in a modified Murashige and Skoog (1962) medium supplemented with 20 μM silver thiosulfate and 4 g/L activated charcoal (named after MS/STS/AC medium), conditions that induced secondary embryogenesis, as described by Martínez et al. (2015). *Phytophthora cinnamomi* strain 1630 was kindly provided by Dr. P. Abad (group Phytopathogenic fungi, Instituto Agroforestal Mediterráneo - Universidad Politécnica de Valencia, Spain) and was maintained in PDA medium (Potato Dextrose Agar, Pronadisa, Spain) by subculturing mycelium pieces of 0.5 cm^2^ to fresh medium every 15 days.

### 2.2 Elicitation and inoculation treatments

Three aliquots of about 1 g of plant material containing somatic embryos at the globular stage were cultured for 3 days in 250 mL Erlenmeyer flasks containing 40 mL of Elicitin Secretion Medium supplemented or not with 50 µM MeJA, as described in Morcillo et al. (2020). After this period, plant material was recovered by filtering and transferred to MS/STS/AC medium.

Suspensions of *P. cinnamomi* strain 1630 were prepared by inoculating in flasks with 40 mL of ESM, 5 sections of 0.5 cm^2^ of active mycelium, taken from 10-days-old PDA cultures. Flasks were then incubated under agitation (50 rpm) and in the dark, in a growth chamber at 23 ± 2 °C for 4 days. Subsequently, oomycete cultures were filtered (Whatman ^®^ paper N2) and the liquid extract was diluted to 20% (v/v) with ESM. For inoculation, samples (1g) of control and elicited embryogenic material, cultivated in MS/STS/AC medium for 5 days, were immersed for 3 h in 40 mL of this oomycete suspension, recovered by filtering and transferred again to solid plates containing MS/STS/AC medium. After 24 h, plant material was stored at -80 °C and lyophilised until analysis. For each treatment: control (C), elicited (MeJA), infected with *P. cinnamomi* (INC), and elicited and infected (MeJA+INC), 3 replicates were prepared.

### 2.3 Protein extraction and peptide extracts preparation

Protein extraction was carried out using the TCA/Acetone method described by Valero Galván et al. (2014) with modifications. Briefly, 0.1 g of lyophilised material was extracted with 10% (w/v) TCA/acetone, 0.07% (w/v) dithiothreitol (DTT) solution. Samples were sonicated (3×10 s, 50 W, amplitude 60, 4°C) and precipitated overnight. The resulting pellets after centrifugation (15000 × g, 4°C, 15 minutes) were washed twice using acetone solution with 0.07% (w/v) DTT, centrifuged again (15000 × g, at 4°C, 15 min) and resuspended in a solubilisation buffer (urea 7M, thiourea 2M, CHAPS 4% (3-[(3-cholamidopropyl) dimethylammonium]-1-propanesulfonate), Tween-20 2%). Protein concentrations were determined using bovine serum albumin (BSA) as standard (Bradford, 1976). Protein extracts were stored at -20 °C until further analysis.

Protein extracts were purified by SDS-PAGE (Laemmli, 1976) following the protocol described in Valledor et al. (2014), using 100 μg of BSA protein equivalents. The sole resulting bands were visualised (Mathesius et al., 2003), digested with trypsin (Promega, Madison, WI) and pre-filtered (300 μm x 5 µm Acclaim Pepmap, Thermo Scientific).

### 2.4 nLC-MS/MS analysis

Tryptic peptides were subjected to nLC-MS/MS at the Proteomics Facility for Research Support Central Service (SCAI) of the University of Cordoba (Spain), using a Dionex Ultimate 3000 nano-LC instrument (Thermo Scientific, CA, USA) coupled to a nanoelectrospray ionisation source and a trihybrid analyser Thermo Orbitrap Fusion (Q-OT-qIT, Thermo Scientific) mass spectrometer. Ten micrograms of tryptic peptides were loaded into a one-dimensional nano-flow LC, peptide separation was performed with a C18 Acclaim Pepmap column (Thermo Scientific) and resolved in 85 min gradient from 5 to 95% of mobile phase B (90% ACN and 0.1% formic acid). Eluting peptides were ionised by nano electrospray ionisation and analysed on the mass spectrometer operated in the positive mode. Peptide precursor scanning from 400 to 1500 m/z was performed at 120K resolution (at 200 m/z) with a counting target of 4 × 105 ions. Tandem MS was performed by isolation at 1.2Da with the quadrupole, CID fragmentation with a normalised collision energy of 35, and MS analysis with fast ion trap scanning. The target AGC ion count was set at 2 x 103 and the maximum injection time was 300 ms. Only those precursors in charge state 2–5 were sampled by MS2. The dynamic exclusion duration was set at 15 s with a tolerance of 10 ppm around the selected precursor and its isotopes. Monoisotopic precursor selection was activated. The equipment operated in top 30 mode with 3 s cycles.

### 2.5 Data processing, protein identification and functional characterisation

Spectra were processed by using the Proteome Discoverer™ version 2.1.0.81 software (Thermo Scientific). MS2 spectra were searched with SEQUEST algorithm against the translated *Q. ilex* transcriptome generated by Guerrero-Sánchez et al. (2019) with searching parameters described by Castillejo et al. (2020): precursor mass tolerance 10 ppm and fragment ion mass tolerance 0.1 Da. Identification confidence was set to FDR ≤ 0.01, variable modifications to oxidation of methionine and fixed modifications to carbamidomethyl cysteine formation. A maximum of two missed cleavages were allowed in each search. Proteins were filtered using more than 1 unique peptide criteria and quantified using peak intensity values normalising by sum and log 2 transformed. After preliminary clustering (Partial Least Squares – Discriminant Analysis, sPLS-DA) and Kruskal Wallis analysis using MetaboAnalyst 5.0 platform, statistically differential proteins were categorised and represented using MapMan4 tools (Thimm et al., 2004; Schwacke et al., 2019) with *Arabidopsis*, PlantDB and *Medicago* as database. The complete proteomics dataset, including raw files, has been deposited in the ProteomeXchange Consortium via the PRIDE repository under the accession number PXD041234.

## 3 Results

### 3.1 Untargeted nLC-MS/MS proteomic profiling

Elicitation with 50 µM MeJA did not affect the external phenotype of the holm oak E00 embryogenic line studied here (**Supplementary Figure 1**), as nor did the further inoculation with *P. cinnamomi* extract at the time that proteins protein profile was studied.

**Figure 1:**
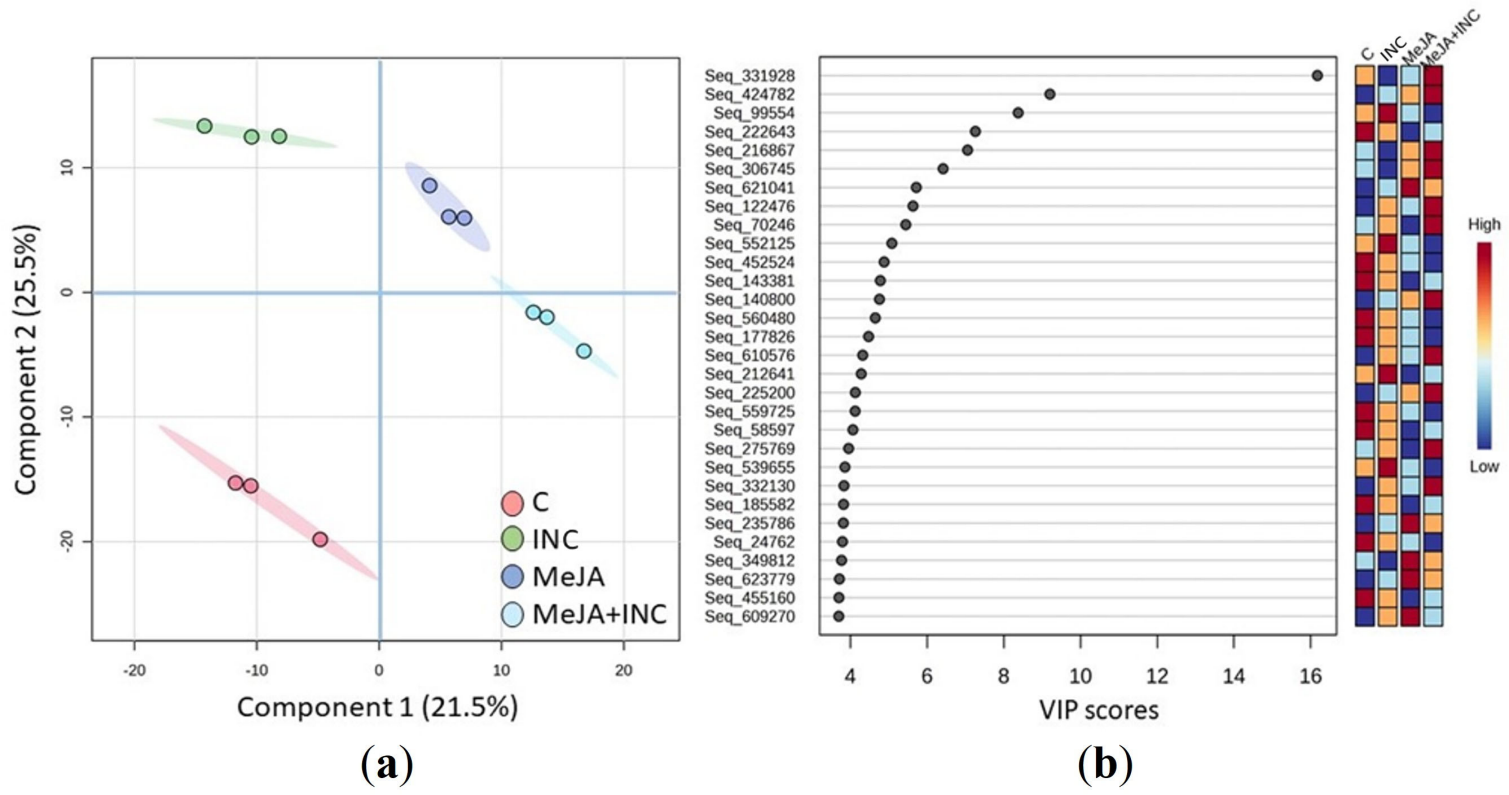
Proteome data analysis. **(a)** PCA of the variable features dataset identified in holm oak E00 embryogenic lines during four treatments (control (C), 3 days with 50 µM methyl-jasmonate (MeJA), 3 hours *Phytophthora cinnamomi* inoculation (INC) and 3 days with 50 µM MeJA + 3 hours *Phytophthora cinnamomi* inoculation (MeJA+INC)); **(b)** Loading plot of the top VIP 30 proteins and heat-map of normalised peak intensities values.

A total of 3,205 protein species were detected in *Q. ilex* samples after filtering (protein species with >20% of coverage, 1 unique peptide and 2 or more matched peptides with our database) using LC-MS/MS. To corroborate the reproducibility and biological influence, sPLS-DA was performed. Three components explained a total of 68.9% of the variance, which split in 21.3%, 25.5% and 22.1% for the PC1, PC2 and PC3 respectively. Biological samples were well grouped into the different treatments applied, being the Control (C) the most separated one. Using the PC3, we observed also a separation between non-infected groups (C and MeJA) and infected cultures (INC and MeJA+INC groups) (**Figure 1a**). Looking at the loading plot to identify 30 proteins that have the largest effect on each component, we represented in a heat-map the values of their normalised peak intensities (**Figure 1b**). Abundances of these protein species, which, as described below, have relevant roles in both inoculation and/or defence mechanisms, did not show a common pattern of variation among the four treatments studied.

**Figure 2:**
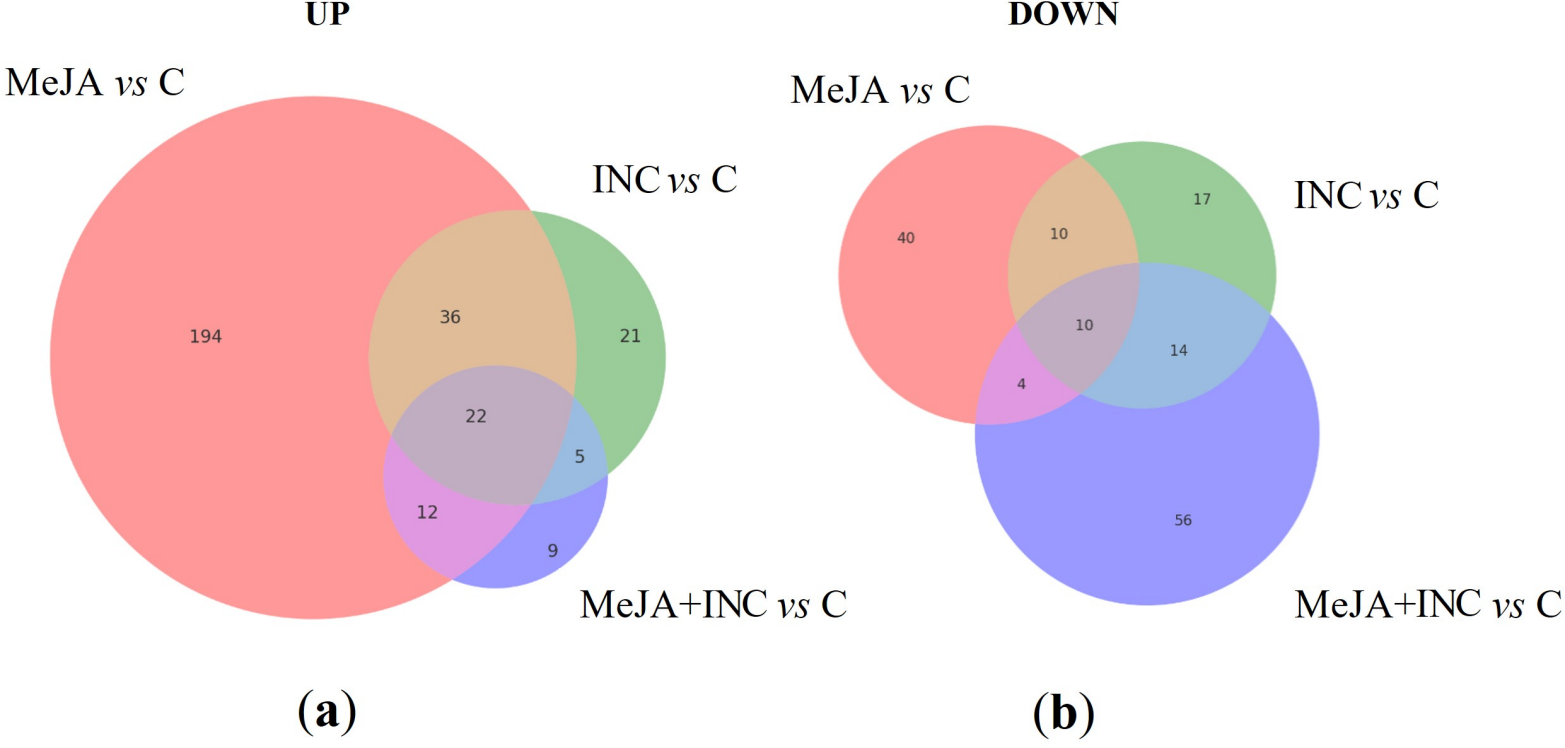
Venn diagram of the number of differentially accumulated proteins with **(a)** fold-change > 2 (UP) or **(b)** fold change < -2 (DOWN) with FDR p ≤ 0.01 in cultures of the *Quercus ilex* E00 embryogenic line subjected to MeJA elicitation, infected with *P. cinnamomi* (INC) or elicited and infected (MeJA+INC) as compared to control embryogenic cultures.

After spotting the major variations by using Mercator v4 tools and MapMan v.3.7.0, we estimated the fold changes for MeJA, INC and MeJA+INC treatments (log2 of the ratios respect to the control group) of the 3,205 proteins. We found more proteins with up-regulated level of expression in MeJA elicited samples than in infected samples (**Figure 2a**), while elicitation induced more proteins to be down-regulated than inoculation (**Figure 2b**).

**Figure 3:**
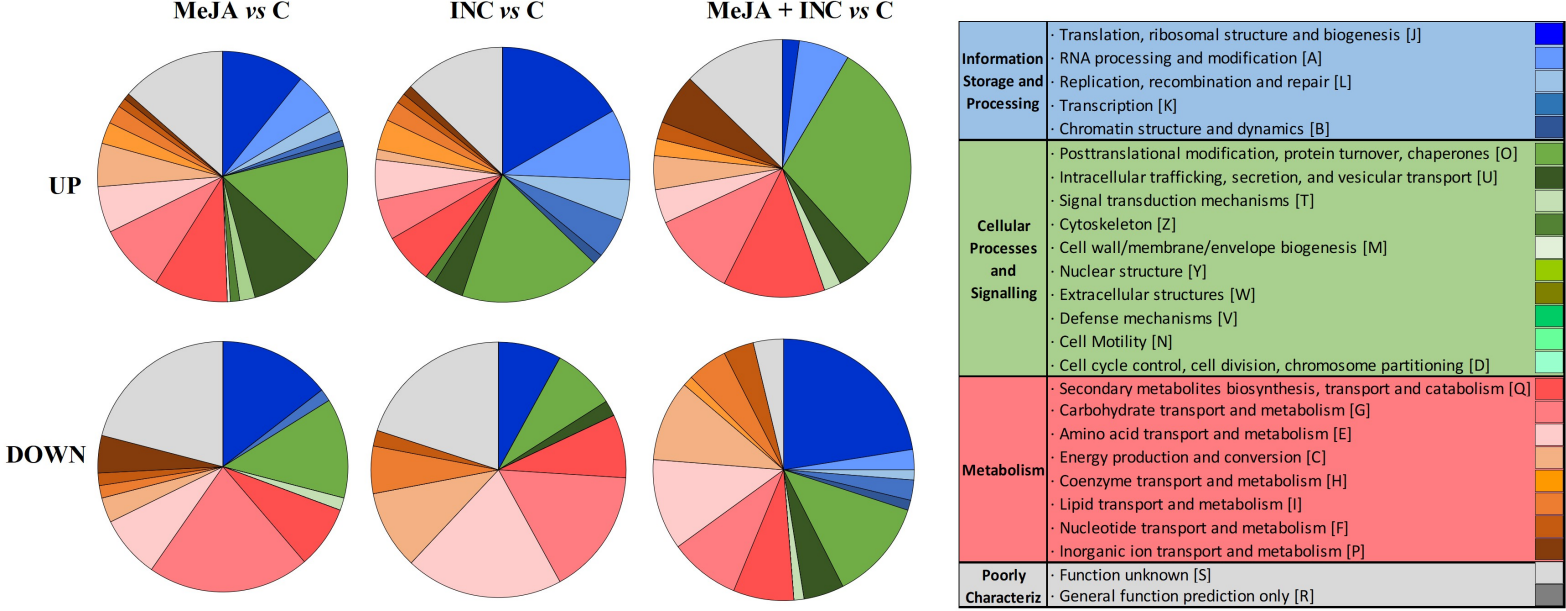
Clusters of Orthologous Groups of proteins (COGs) derived from the analysis of function categories with more variable transcripts observed when comparing MeJA *vs* C, INC *vs* C and MeJA+INC *vs* C E00 embryogenic line from holm oak. The four COG categories and 25 subcategories were analysed as: Information storage and processing (J, A, L, K and B), Cellular processes and signalling (O, U, T, Z, M, Y, W, V, N and D), Metabolism (Q, G, E, C, H, I, F and P) and Poorly characterised (S and R).

Gene ontology analysis showed the overview of the proteins differentially accumulated in holm oak embryogenic lines after MeJA elicitation and/or inoculation with *P. cinnamomi* (**Figure 3**), and particularly of those related with biotic stress (**Supplementary Figure 2**). Among these proteins, we found higher accumulation of those related to hormone signalling in the MeJA+INC group than in MeJA or INC groups. Regarding the general metabolism, MeJA treatment induced over-accumulation of proteins, especially those related with cell wall biogenesis, secondary metabolism and amino acid metabolism (**Supplementary Figure 3**). This protein over-accumulation was less pronounced in the MeJA+INC group. Finally, infected embryogenic cell lines (INC) were characterised by under-accumulation of secondary metabolism-related proteins.

**Figure 4:**
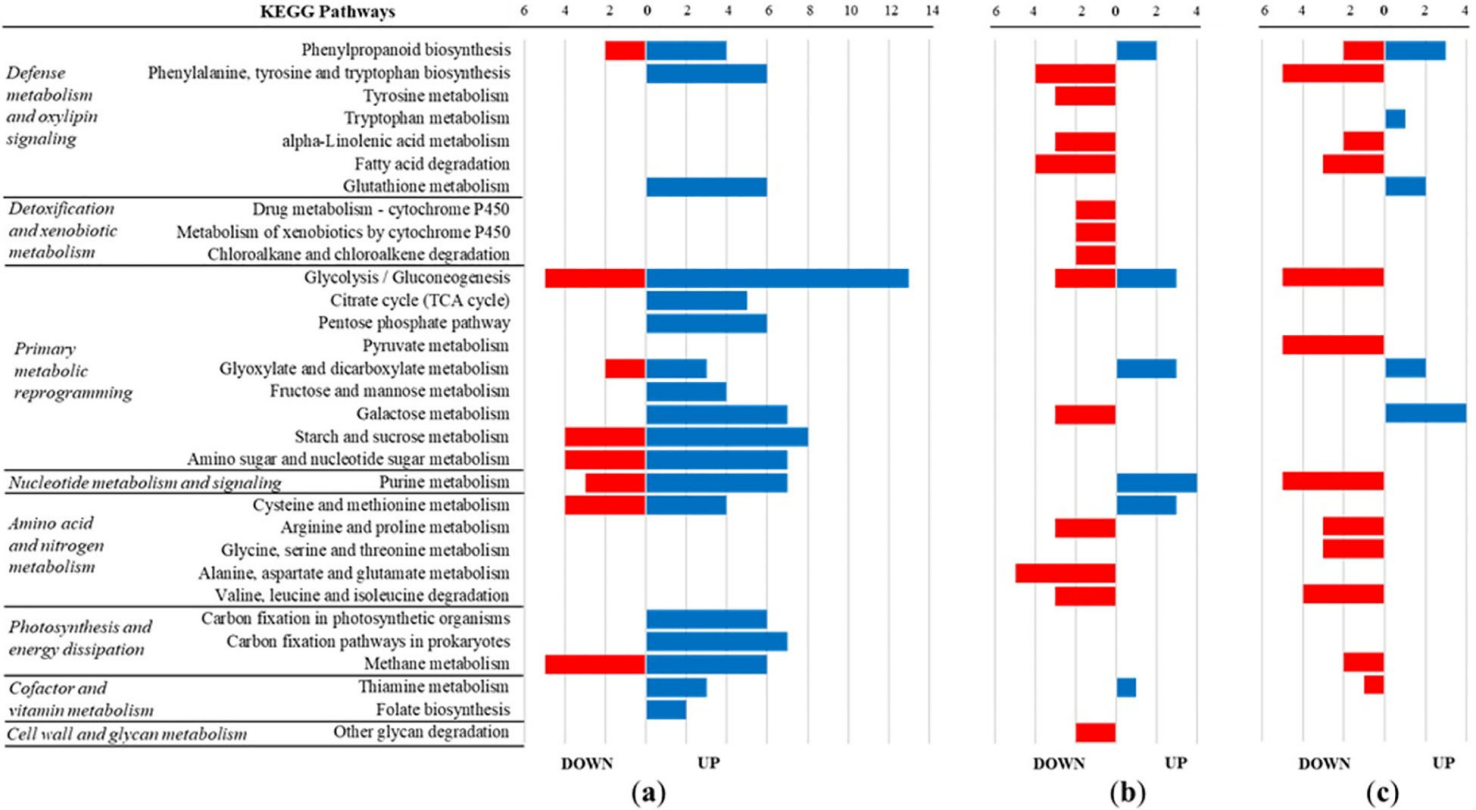
Distribution of up- and downregulated enzymes among KEGG-based functional categories under **(a)** MeJA elicitation (MeJA *vs* C), **(b)***P. cinnamomi* inoculation (INC *vs* C), and **(c)** combined MeJA elicitation and inoculation (MeJA + INC *vs* C). Enzymes were classified according to their putative function based on the Kyoto Encyclopedia of Genes and Genomes (KEGG). The number of enzymes within each category reflects differential representation relative to the control condition.

Holm oak embryogenic cells treated with MeJA showed increased abundance of enzymes involved in carbohydrate metabolism and in the biosynthesis of amino acids and nucleotides, indicating a global metabolic activation (**Figure 4a**). The MeJA elicitation treatment markedly enhanced pathways related to glycolysis/gluconeogenesis, starch and sucrose metabolism, and photosynthesis and energy dissipation, while moderately stimulating enzymes associated with defence and oxylipin metabolism. In contrast, cells infected with *P. cinnamomi* displayed a general reduction in enzyme representation, particularly in amino acid, nitrogen, and detoxification/xenobiotic metabolism (**Figure 4b**). A comparable pattern was observed when inoculation occurred in MeJA-treated cells (**Figure 4c**), with a strong repression of core carbon and amino acid metabolism. Nevertheless, enzymes involved in phenylpropanoid and glutathione metabolism remained selectively up-regulated, suggesting the maintenance of specific antioxidant and phenolic defence functions under combined stress.

### 3.2 Conserved defence-responsive proteins under MeJA and inoculation

Comparison of **Figures 4a, b** reveals that both MeJA elicitation and pathogen inoculation share the activation of the phenylpropanoid biosynthetic pathway. In addition, purine and thiamine metabolism, as well as the glyoxylate cycle (involved in lipid turnover in plants), were commonly up-regulated under both conditions.

Elicitation and inoculation treatments performed in holm oak embryogenic lines increased the abundance of 22 proteins and decreased the abundance of 10 proteins (**Table 1**). These treatments resulted in higher levels of proteins related with the phenolics metabolism such as cinnamate-4 hydroxylase and caffeoyl-CoA O-methyltransferase, as well as enzymes that regulate the glutathione balance, and other related with stress response (calreticulin, thaumatin-like protein). Besides, increased levels of regulatory proteins involved in protein synthesis and degradation were observed, while some others showed significant down-regulation.

**Table 1:** Proteins differentially accumulated using the control treatment as reference in holm oak E00 embryogenic lines subjected to methyl-jasmonate elicitation and/or inoculation with *Phytophthora cinnamomi* (MeJA, INC, and MeJa+INC treatments).

	Sequence	Annotation
UP	Seq_115047	Cinnamate-4-hydroxylase
	Seq_117573	Caffeoyl-CoA O-methyltransferase
	Seq_131712	60S ribosomal protein L28-1
	Seq_135140	Putative ATP-dependent zinc metalloprotease FTSH 8, mitochondrial-like
	Seq_147526	Tau class glutathione S-transferase
	Seq_166419	E3 ubiquitin-protein ligase UPL1-like protein
	Seq_22311	Glycine cleavage system H protein 2, mitochondrial
	Seq_240202	Inosine-5’-monophosphate dehydrogenase
	Seq_256860	RNA and export factor binding protein, putative
	Seq_262728	Calreticulin
	Seq_309412	Hemoglobin-2
	Seq_331738	Tau class glutathione S-transferase
	Seq_361460	Putative mannitol dehydrogenase
	Seq_372631	Alcohol dehydrogenase
	Seq_490730	Serine/arginine-rich splicing factor RS31A
	Seq_508565	G-Rich RNA Sequence binding Factor (GRSF)
	Seq_564203	NEDD8-activating enzyme E1 catalytic subunit
	Seq_59184	S-adenosylmethionine synthase
	Seq_605051	GEM-like protein 5
	Seq_612593	Bifunctional inhibitor/lipid-transfer protein/seed storage 2S albumin superfamily protein
	Seq_7992	Thaumatin-like protein
	Seq_99664	Short chain alcohol dehydrogenase, putative
DOWN	Seq_7227	Alcohol dehydrogenase
	Seq_404598	40S ribosomal protein S20-2
	Seq_505400	Ubiquitin-conjugating enzyme E2 10
	Seq_152548	Arginase
	Seq_440991	Putative glycosyltransferase At1g55740 family
	Seq_374843	Translation elongation factor 1
	Seq_273373	Inositol monophosphatase 1
	Seq_240383	NADH dehydrogenase [ubiquinone] 1 alpha subcomplex subunit 2
	Seq_547157	Glyoxalate Reductase 1GLYR1
	Seq_123987	Branched-chain-amino-acid aminotransferase-like protein 2

(UP >2n-fold and DOWN <-2n-fold).

Inoculation with *P. cinnamomi* of MeJA-treated holm oak embryogenic cells resulted in over-abundance of 23 proteins when compared with levels induced by oomycete inoculation in non-elicited cells (**Table 2**). Some of these proteins are involved in cell response to stress, such as those regulating glutathione, as well as catalase, thaumatin-like protein and chitinase.

**Table 2:** Proteins differentially accumulated in MeJA elicited holm oak embryogenic lines as compared to non-elicited plant material, after inoculation with *P. cinnamomi* (MeJA+INC) – (INC).

Sequence	Annotation	Accumulation
Carbohydrates, cell wall and energy metabolism		
Seq_347385	Alpha-mannosidase	UP
Seq_288988	Xyloglucan endotransglucosylase/hydrolase	UP
Seq_467706	UDP-glucose 4-epimerase	UP
Seq_16777	Vacuolar invertase	UP
Seq_117051	Beta-galactosidase	UP
Synthesis, degradation and protein folding metabolism		
Seq_476585	Asparagine synthetase B	UP
Seq_343265	Cysteine protease-like protein	UP
Seq_329399	Carboxypeptidase	UP
Seq_204426	Putative uncharacterised protein PDI	UP
Seq_416565	Importin subunit alpha	UP
Seq_507904	Putative metallophosphatase	UP
Seq_394736	Gamma-soluble NSF attachment protein	UP
Signalling and transport		
Seq_121841	V-type proton ATPase subunit a	UP
Redox activity		
Seq_13751	Probable glutathione peroxidase 2	UP
Seq_552114	Catalase	UP
Seq_396761	Glutathione S-transferase isoform 2	UP
Seq_54038	Reticuline oxidase-like protein	UP
Seq_199066	Short chain alcohol dehydrogenase, putative	UP
Seq_464912	Germin-like protein subfamily 2, member 4	UP
Pathogen responses		
Seq_167836	Thaumatin-like protein	UP
Seq_70246	Basic chitinase type I	UP
Carbohydrates and energy metabolism		
Seq_228702	ATP-PFK	DOWN
Seq_257619	Glyceraldehyde-3-phosphate dehydrogenase	DOWN
Seq_255678	Pyruvate kinase	DOWN
Seq_239199	Phosphofructokinase 6	DOWN
Seq_588655	Malic enzyme	DOWN
Seq_586166	Oxoglutarate dehydrogenase (Succinyl-transferring), E1 component	DOWN
Seq_514234	Acetyl-CoA C-acyltransferase	DOWN
Seq_589638	Acetoacetyl-CoA thiolase	DOWN
Seq_365163	NADH dehydrogenase	DOWN
Seq_309918	NADH dehydrogenase [ubiquinone] 1 alpha subcomplex subunit 13-A	DOWN
Secondary metabolism and defence		
Seq_262308	Cinnamyl alcohol dehydrogenase	DOWN
Seq_589557	Bifunctional 3-dehydroquinate dehydratase/shikimate dehydrogenase, chloroplastic	DOWN
Seq_209322	(+)-neomenthol dehydrogenase	DOWN
Seq_573553	Spermine synthase	DOWN
Seq_244900	Bifunctional 3-dehydroquinate dehydratase/shikimate dehydrogenase, chloroplastic	DOWN
Seq_470004	Glycine-rich protein 2b	DOWN
Seq_269850	Putative glycosyltransferase At1g55740 family	DOWN
Seq_333685	Putative glycosyltransferase At1g55740 family	DOWN
Seq_27270	Putative glycosyltransferase At1g55740 family	DOWN
Seq_14690	Chitinase	DOWN
Seq_162185	Thaumatin-like protein	DOWN
Seq_157660	Non-specific lipid-transfer protein	DOWN
Seq_331928	Alpha amylase/subtilisin inhibitor	DOWN
Seq_531108	Endo-1,31,4-beta-D-glucanase	DOWN
Redox activity		
Seq_153381	Mitochondrial acidic protein MAM33, mitochondrial, putative	DOWN
Seq_116247	APX2	DOWN
Protein Synthesis, degradation, modification and folding		
Seq_27198	Heat shock protein 90	DOWN
Seq_295649	3-hydroxyisobutyryl-CoA hydrolase-like protein 3, mitochondrial	DOWN
Seq_481610	Stress-induced-phosphoprotein 1	DOWN
Seq_604863	Tryptophan synthase beta chain	DOWN
Seq_14146	Protein-lysine N-methyltransferase AMTR_s00105p00145330	DOWN
Seq_38224	Protease Do-like 7	DOWN
Seq_552426	Calnexin-like protein 1	DOWN
Seq_354032	T-complex protein 1 subunit gamma	DOWN
Seq_333060	T-complex protein 1 subunit beta	DOWN
Seq_79003	Putative prefoldin subunit 5	DOWN
Seq_108314	Ubiquitin-conjugating enzyme E2 27	DOWN
Seq_600165	Importin subunit alpha	DOWN
Seq_212498	Glycine cleavage system P-protein	DOWN
Structural, transport and signalling		
Seq_502930	Vesicle-fusing ATPase	DOWN
Seq_327503	Microtubule-associated protein MAP65-1a	DOWN
Seq_98449	Ras-related protein Rab11C	DOWN
Seq_588567	Putative voltage-gated potassium channel subunit beta	DOWN
Seq_219248	Mitochondrial ornithine transporter 1	DOWN
Translation, gene expression and DNA/RNA processing		
Seq_63240	Cell division cycle 5-like protein	DOWN
Seq_566258	Histone deacetylase HDT1	DOWN
Seq_89758	Histone deacetylase HDT1	DOWN
Seq_363591	Polyadenylate-binding protein RBP47B	DOWN
Seq_7758	40S ribosomal protein S5	DOWN
Seq_181098	40S ribosomal protein S14-1	DOWN
Seq_511859	40S ribosomal protein S2-4	DOWN
Seq_517356	40S ribosomal protein S11-1	DOWN
Seq_259672	60S ribosomal protein L22-2	DOWN
Seq_560480	40S ribosomal protein S5	DOWN
Seq_201170	40S ribosomal protein S15	DOWN
Seq_252678	40S ribosomal protein S3-1	DOWN
Seq_281238	60S ribosomal protein L10	DOWN
Seq_88357	60S ribosomal protein L13a-2	DOWN
Seq_429823	Probable 40S ribosomal protein S3	DOWN
Seq_171660	40S ribosomal protein S17	DOWN
Seq_379336	Histone-binding protein RBBP4	DOWN
Seq_248213	Small nuclear ribonucleoprotein-associated protein B	DOWN
Seq_519759	Zinc finger CCCH domain-containing protein 14	DOWN
Seq_215662	Nucleoside diphosphate kinase 1	DOWN
Seq_320314	Aminoacyl tRNA synthase complex-interacting multifunctional protein 1	DOWN
Seq_570670	Rossmann-fold NAD(P)-binding domain-containing protein	DOWN
Seq_9770	Dehydrogenase/reductase SDR family member 4	DOWN
Seq_247180	N-acetyl-gamma-glutamyl-phosphate reductase	DOWN
Seq_111031	Eukaryotic translation initiation factor 3–93 kDa subunit homolog	DOWN
Seq_190285	DNA helicase	DOWN
Seq_503774	Coatomer subunit alpha	DOWN
Seq_505778	Putative aldehyde dehydrogenase	DOWN
Seq_376999	Protein TOC75, chloroplastic	DOWN
Seq_354139	Deoxyuridine 5’-triphosphate nucleotidohydrolase	DOWN
Seq_325643	Phospho-2-dehydro-3-deoxyheptonate aldolase	DOWN
Seq_381520	AMPSase	DOWN
Seq_499311	Ankyrin repeat-containing protein	DOWN
Seq_107922	Leucyl-tRNA synthetase	DOWN
Seq_574938	Alcohol dehydrogenase C	DOWN
Unknown or uncharacterised		
Seq_559311	Os05g0110300 protein	DOWN
Seq_151542	Unknown protein	DOWN
Seq_365961	Uncharacterised protein At5g02240	DOWN
Seq_511906	At2g15220/F15A23.4	DOWN
Seq_102867	BnaA10g21320D protein	DOWN

(UP >2n-fold and DOWN <-2n-fold).

### 3.3 Proteomic profile associated with MeJA priming during inoculation

A total of 106 proteins showed significant differential accumulation (p < 0.05) between *P. cinnamomi*-infected holm oak embryogenic lines pre-treated with MeJA and the non-elicited infected controls (**Table 2**). Among these, 85 proteins (80.2%) were down-accumulated, and 21 proteins (19.8%) were up-accumulated after MeJA priming. Functional classification revealed that the differentially abundant proteins were mainly associated with primary metabolism (37 proteins), stress and defence responses (30 proteins), protein folding and trafficking (17 proteins), oxidation–reduction processes (12 proteins), and signalling or regulatory functions (11 proteins).

Down-accumulated proteins were predominantly involved in energy and carbohydrate metabolism, including enzymes such as phosphofructokinase, pyruvate kinase, and malic enzyme, as well as components of ribosomal and translation machinery, indicating a general reduction of primary metabolic activity and protein synthesis under MeJA priming during inoculation. Conversely, the up-accumulated proteins included key defence-related enzymes such as basic chitinase type I, thaumatin-like protein, germin-like protein, catalase, glutathione peroxidase, and glutathione S-transferase isoform 2, together with proteins related to cell wall remodelling (e.g., xyloglucan endotransglucosylase/hydrolase) and carbohydrate metabolism (vacuolar invertase, β-galactosidase). These findings suggest that MeJA priming enhances the antioxidant and pathogenesis-related (PR) defence machinery, while downregulating energy-demanding biosynthetic processes.

## 4 Discussion

Previously we reported that elicitation of holm oak embryogenic lines with 50 µM MeJA did not affect their further growth and development, while induced direct defence responses based on an increase in ABA and JA biosynthesis, and accumulation of phenolic compounds. In addition, after challenged against *P. cinammomi*, using a dual culture approach, elicited embryogenic material displayed a defence profile characterised by a further increase in JA, slightly inhibition of mycelia growth, and higher hydrogen peroxide production (Morcillo et al., 2020; 2022). To enlighten the mechanisms underlying this phenotype we studied the proteomic profile induced by MeJA before and after oomycete inoculation.

The proteomic response of holm oak (*Quercus ilex*) embryogenic lines to *P. cinnamomi* inoculation (INC) and/or methyl jasmonate (MeJA) elicitation revealed key insights into the defence-related metabolic reprogramming associated with biotic stress responses. Overall, *P. cinnamomi* inoculation led to a general down-regulation of protein abundance, consistent with previous studies in other plant species subjected to pathogenic stress (Sghaier-Hammami et al., 2021). This trend suggests suppression or redirection of core cellular metabolism during inoculation, possibly due to the pathogen’s manipulation of host physiological processes or the redirection to defence mechanisms like antioxidant compounds production. For example, our data pattern (**Figure 2**) is consistent with the expected effects of pathogen inoculation. Changes in metabolite levels often reflect either direct effects of inoculation or the host’s induced defence response. In general, decreases are interpreted as metabolic effects of pathogen activity or resource depletion, whereas increases are typically associated with host defence or priming responses (Bolton, 2009).

A key challenge in mitigating the effects of *P. cinnamomi* is the identification of molecular and physiological markers that differentiate resistant from susceptible genotypes. The constitutive expression of defence genes and the speed of defence activation are crucial indicators of resistance, as shown in *Castanea crenata*, which displays faster responses than the more susceptible *C. sativa* (Serrazina et al., 2015). Similarly, in avocado, early callose deposition at infection sites effectively limited *P. cinnamomi* penetration, underscoring the importance of early cellular defences in resistance (Coelho et al., 2021). Moreover, a recent multiomic approach (Triviño et al., 2025) reports on the identification of 29 candidate genes to be used as molecular markers to select holm oak genotypes with a better adaptation to drought and *P. cinnamomi* inoculation, both producing the species decline.

The metabolic profiles obtained indicate distinct and complementary effects of MeJA elicitation and *P. cinnamomi* inoculation. MeJA alone promoted an extensive metabolic reprogramming, reinforcing energy-producing pathways and JA-dependent defence mechanisms, consistent with the establishment of a primed defensive state (Bertini et al., 2019; Mageroy et al., 2020). In contrast, inoculation by *P. cinnamomi* caused a general metabolic downshift, possibly reflecting a pathogen-induced restriction of host metabolic activity and resource allocation as described in Cai et al. (2011). When both stimuli were combined, MeJA priming attenuated the inoculation-induced suppression by maintaining the activation of key defence-related routes, particularly phenylpropanoid and glutathione metabolism as described by Ji et al. (2021) and Liu et al. (2023). These findings suggest that MeJA preconditioning enables holm oak cells to prioritise antioxidant and phenolic defences during pathogen challenge, partially compensating for the broad repression of primary metabolism caused by inoculation.

In our experimental setting, MeJA elicitation promoted the accumulation of a higher number of proteins, particularly those related to phenylpropanoid biosynthesis (e.g., cinnamate-4-hydroxylase and caffeoyl-CoA O-methyltransferase), stress signalling (e.g., Stress-induced-phosphoprotein 1, Heat Shock Protein 90), pathogen related (e.g., thaumatin-like proteins, basic chitinase type I), and redox homeostasis (e.g., calreticulin, glutathione-related enzymes). These changes reflect the central role of jasmonate signalling in orchestrating induced defences and secondary metabolite pathways that contribute to pathogen resistance (Wasternack and Song, 2017). The accumulation of cinnamate-4-hydroxylase, that catalyses the second step of the phenylpropanoid pathway to generate *p-*coumaric acid, the precursor of numerous phenolic compounds (Khatri et al., 2023), might account for the higher content in phenolic acids previously reported for the MeJA-elicited holm oak embryogenic lines (Morcillo et al., 2022). In turn, S-adenosylmethionine synthase and caffeoyl-CoA O-methyltransferase are responsible for methylation of the hydroxy groups of flavonoids (Liu et al., 2023).

Interestingly, the MeJA+INC group did not synergistically amplify this protein accumulation and, in some cases, showed a diminished response compared to MeJA alone (e. g., chitinase and thaumatin-like protein). This suggests that while MeJA pre-conditioning primes the cells for defence, the pathogen may interfere with or override certain aspects of this priming upon inoculation. Notably, two PR identified proteins were over-accumulated in the MeJA+INC samples, the basic chitinase type I, protein that target and degrade chitin, which is the main structural part of cell walls of the pathogenic fungi (Durand et al., 2021), and thaumatin-like protein that confers enhanced resistance to a broad spectrum of phytopathogenic fungi (Hejgaard et al., 1991; Grenier et al., 1999) because of their ability to change the permeability of cell membranes and inhibit spore germination and mycelial growth of the target fungi (Jiao et al., 2018; Zhao et al., 2023). Despite the lack of chitin in oomycetes, there are some studies that characterised these genes in *Phytophthora* spp (Hinkel and Ospina-Giraldo, 2017). Note, however, that some isoforms of both proteins were under accumulated (**Table 2**). It is worth noting that in bottom-up proteomics, protein identification often relies on conserved peptide regions, which can match multiple isoforms or members of multigene families, even if they differ in expression or substrate specificity (Dupree et al., 2020; Miller et al., 2022). The observation that some defence protein isoforms were over−accumulated while others were under−accumulated following methyl jasmonate (MeJA) treatment can be explained by both biological regulation and technical factors. Many defence proteins, such as chitinases (PR-3 family) and thaumatin-like proteins (P-5 family), are encoded by large multigene families whose members are differentially regulated by hormonal signalling pathways; thus, MeJA induces specific chitinase isoforms involved in jasmonate-mediated defence responses whereas other PR proteins can be constitutively expressed or more responsive to salicylic acid (SA) signalling (Sabater-Jara et al., 2011; van Loon et al., 2006). The antagonistic crosstalk between JA and SA pathways is well documented in *Arabidopsis*, where SA strongly suppresses expression of many jasmonate-responsive genes, providing a mechanistic basis for why some defence isoforms may be repressed even under JA/MeJA treatment (Pieterse et al., 2012). From a technical perspective, bottom-up proteomics often infers protein identities based on shared peptide sequences among closely related paralogs, which can mask isoform-specific abundance changes and contribute to apparently contradictory results.

Regarding the response to inoculation of elicited plant material (MeJA+INC), we also observed that protein categories involved in hormone signalling, particularly jasmonate-associated regulators, were more abundant in MeJA+INC samples, indicating partial retention of elicitor-triggered defence signalling under pathogen challenge. This result can be associated to the elevated jasmonic acid content found in MEJA+INC lines in our previous work (Morcillo et al., 2022). Gene ontology and protein functional analyses further supported these findings, highlighting the enrichment of proteins involved in cell wall biogenesis, amino acid metabolism, and secondary metabolism detected in the MeJA treatment. Conversely, the infected group (INC) was characterised by a significant depletion of these functional categories, emphasising the pathogen’s impact on the host’s structural and metabolic defences. This is consistent with reports that *P. cinnamomi* targets host cell wall integrity and metabolism to facilitate penetration and colonisation (Coelho et al., 2021).

The estimation of protein fold changes across the 3,205 identified proteins confirmed a higher number of up-regulated proteins in MeJA-elicited lines than in those only infected, reinforcing the role of jasmonate in pre-activating protective pathways. These results align with findings in other woody species, where jasmonate signalling enhanced resistance through pre-emptive activation of defence genes and proteins (García-Pineda et al., 2010). From a broader perspective, our results highlight the potential of MeJA elicitation as a strategy to prime holm oak tissues against *P. cinnamomi*. Identifying proteins that show significant accumulation upon elicitation or depletion upon inoculation can contribute to the development of molecular markers for resistance screening. For example, consistent over-accumulation of specific secondary metabolism enzymes or redox regulators could serve as early indicators of a primed state. Specifically, the overlap between MeJA elicitation and pathogen inoculation (**Figure 4**) highlights a conserved defence signature, particularly the activation of phenylpropanoid metabolism, which is central to the synthesis of lignin, flavonoids, and other antimicrobial compounds known to fortify structural barriers and restrict pathogen spread (Dixon et al., 2002; Vogt, 2010).

The simultaneous upregulation of purine and thiamine metabolism, together with the glyoxylate cycle, suggests a reprogramming of primary metabolism to support defence-related demands, including nucleotide biosynthesis and energy balance under stress conditions (Bolton, 2009). Interestingly, the consistent induction of glycolysis under both conditions may reflect the need to provide carbon skeletons for secondary metabolite biosynthesis, such as ellagic acid precursors, which have been reported to contribute to antioxidant and antimicrobial defence (Landete, 2011). These shared responses reinforce the idea that elicitor-induced priming and pathogen-triggered immunity converge on core metabolic pathways that underpin effective resistance.

This study contributes to the ongoing search for physiological and molecular markers capable of distinguishing resistant from susceptible genotypes in holm oak (*Quercus ilex*), a keystone Mediterranean species of ecological and agronomic value. The proteomic adjustments observed under MeJA elicitation and *Phytophthora cinnamomi* inoculation underscore the crucial role of hormone signalling, redox regulation, and secondary metabolism in the plant’s defence repertoire. The over-accumulation of pathogenesis-related (PR) proteins, particularly basic chitinase type I and thaumatin-like proteins, reinforces their established antifungal functions through chitin degradation and membrane disruption, mechanisms that collectively inhibit fungal spore germination and mycelial growth (Durand et al., 2021; Zhao et al., 2023; Grenier et al., 1999; Li et al., 2023). Significant up-regulation of chitinase during *P. cinnamomi* root inoculation have been also reported in *Q. suber* (Ebadzad and Cravador, 2014) what suggests the importance of this enzyme in plant defence against the pathogen attack and constitutes probably, an effective response to lessen *P. cinnamomi* damage. These findings align with previous studies in other woody crops, where constitutive defence gene expression and rapid activation of defence responses were critical determinants of resistance, as shown in chestnut (Serrazina et al., 2015); and avocado (Coelho et al., 2021).

Beyond their physiological relevance, these insights carry practical implications for the sustainability of forest and nut crops. Holm oak is not only essential to Mediterranean ecosystems but also supports the acorn-based agri-food sector, where acorns are gaining renewed attention as a nutrient-rich, gluten-free flour alternative with potential for functional food applications. Enhancing resistance to *P. cinnamomi* in holm oak through molecular breeding or biotechnological approaches could therefore preserve genetic resources, safeguard acorn yields, and support the diversification of sustainable plant-based food sources. Altogether, the molecular signatures identified in this work provide valuable insight into the mechanisms of induced resistance in holm oak, and represent promising targets for selecting tolerant genotypes and guiding future breeding and biotechnological strategies that integrate both forest conservation and food innovation goals. In line with integrated pest management strategies, MeJA-induced defence reprogramming represents a preventive approach that can be implemented at early developmental stages in nurseries, complementing genetic and biotechnological interventions aimed at improving long-term resistance.

## Data Availability

The datasets presented in this study can be found in online repositories. The names of the repository/repositories and accession number(s) can be found below: https://www.ebi.ac.uk/pride/archive/, PXD041234.

## Supplementary material

Supplementary Figure 1 Holm oak embryogenic line E00 after elicitation with MeJA. Bar = 1cm.

Supplementary Figure 2 MapMan overview of differentially accumulated proteins related to abiotic or biotic stress in the holm oak E00 embryogenic line after elicitation with methyl-jasmonate and/or inoculation with *Phytophthora cinnamomi*. From left to right: MeJA, INC, and MeJA+INC treatments.

Supplementary Figure 3 MapMan overview of differentially accumulated proteins related to general metabolism in the holm oak E00 embryogenic line after elicitation with methyl-jasmonate and/or inoculation with *Phytophthora cinnamomi*. From left to right: MeJA, INC, and MeJA+INC treatments.
