# Supplementary figures and images for: Understanding MeJA induced-resistance to Phytophthora cinnamomi in holm oak embryogenic lines
Source: Front Plant Sci. 2026 Feb 2;16:1740888. doi: 10.3389/fpls.2025.1740888 (PMC12907432; doi:10.3389/fpls.2025.1740888)

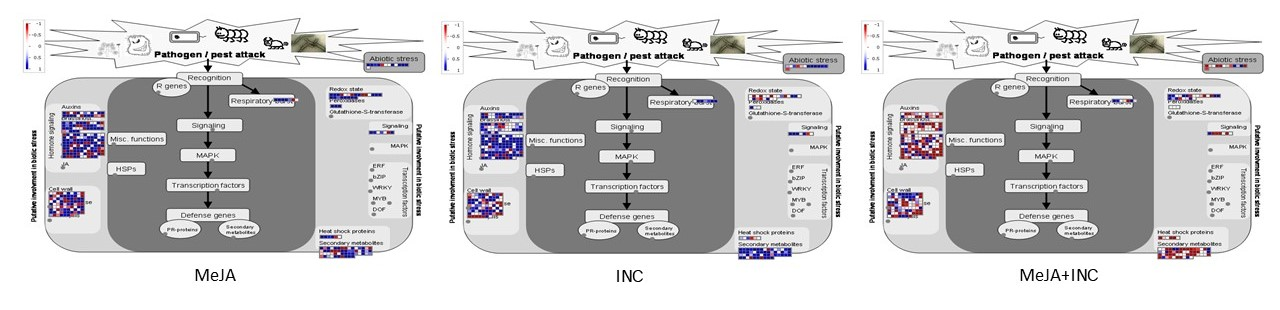

Supplement: Supplementary Figure 1 — Holm oak embryogenic line E00 after elicitation with MeJA. Bar = 1cm. [file Image1.tif]

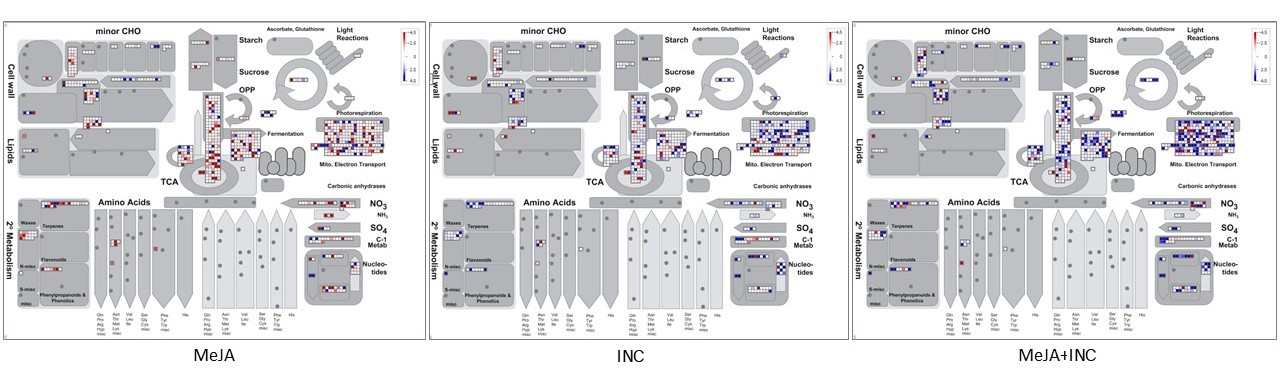

Supplement: Supplementary Figure 2 — MapMan overview of differentially accumulated proteins related to abiotic or biotic stress in the holm oak E00 embryogenic line after elicitation with methyl-jasmonate and/or inoculation with Phytophthora cinnamomi. From left to right: MeJA, INC, and MeJA+INC treatments. [file Image2.tif]

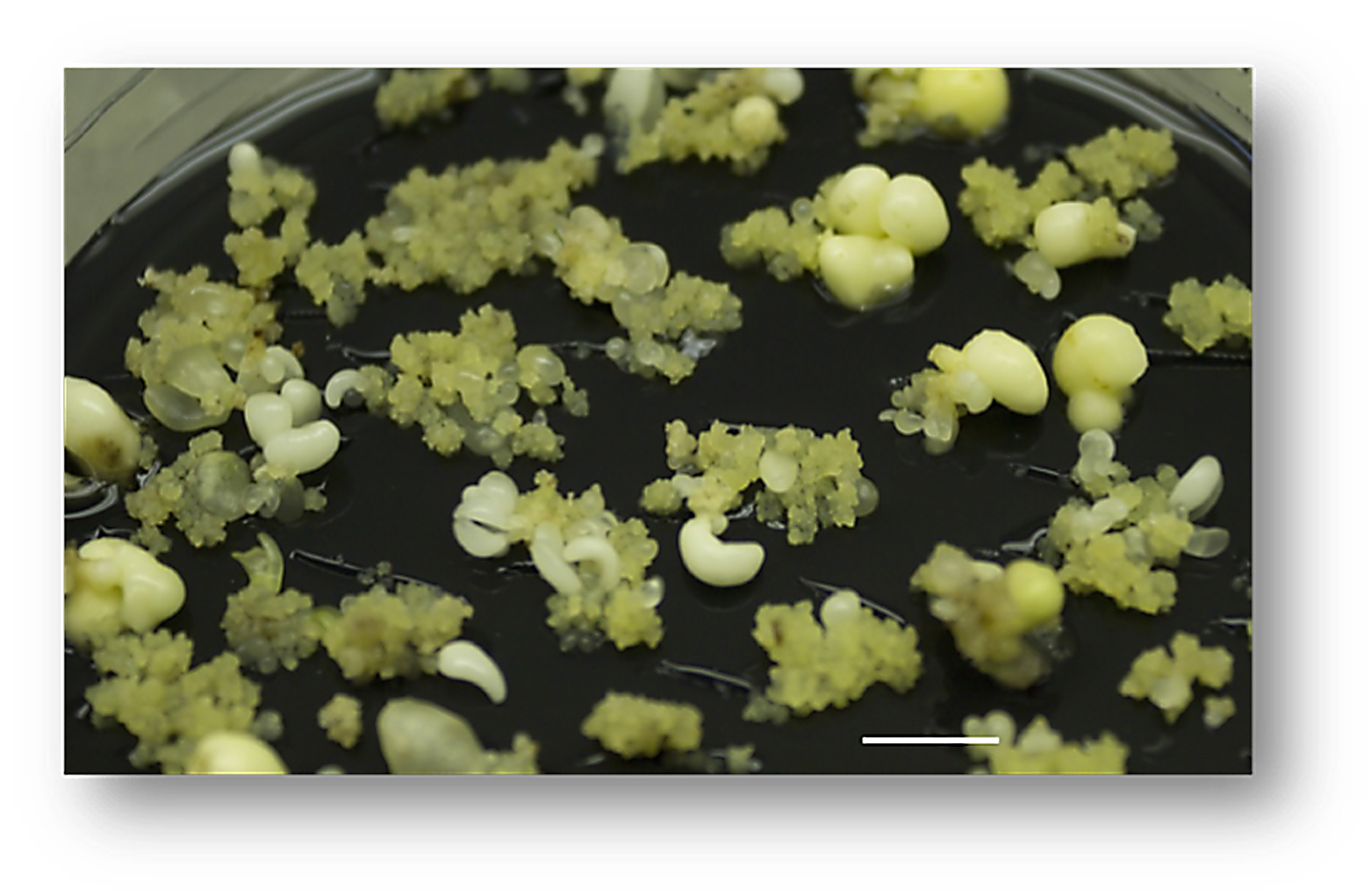

Supplement: Supplementary Figure 3 — MapMan overview of differentially accumulated proteins related to general metabolism in the holm oak E00 embryogenic line after elicitation with methyl-jasmonate and/or inoculation with Phytophthora cinnamomi. From left to right: MeJA, INC, and MeJA+INC treatments. [file Image3.tif]
